# Comparison of Efficacy and Safety Between Laparoscopic and Open Radical Resection for Hilar Cholangiocarcinoma—A Propensity Score-Matching Analysis

**DOI:** 10.3389/fonc.2022.1004974

**Published:** 2022-09-26

**Authors:** Yong-Gang He, Wen Huang, Qian Ren, Jing Li, Feng-Xia Yang, Chang-Lin Deng, Li-Qi Li, Xue-Hui Peng, Yi-Chen Tang, Lu Zheng, Xiao-Bing Huang, Yu-Ming Li

**Affiliations:** ^1^ Department of Hepatobiliary, The Second Affiliated Hospital of Army Medical University, Chongqing, China; ^2^ Department of General Surgery, The Second Affiliated Hospital of Army Medical University, Chongqing, China

**Keywords:** laparoscopic hilar cholangiocarcinoma, open hilar cholangiocarcinoma, retrospective study, propensity score matching, R0 resection

## Abstract

**Background:**

Radical resection remains the most effective treatment for hilar cholangiocarcinoma (HCCA). However, due to the complex anatomy of the hilar region, the tumor is prone to invade portal vein and hepatic arteries, making the surgical treatment of HCCA particularly difficult. Successful laparoscopic radical resection of HCCA(IIIA, IIIB) requires excellent surgical skills and rich experience. Furthermore, the safety and effectiveness of this operation are still controversial.

**Aim:**

To retrospectively analyze and compare the efficacy and safety of laparoscopic and open surgery for patients with HCCA.

**Methods:**

Clinical imaging and postoperative pathological data of 89 patients diagnosed with HCCA (IIIA, IIIB) and undergoing radical resection in our center from January 2018 to March 2022 were retrospectively analyzed. Among them, 6 patients (4 were lost to follow-up and 2 were pathologically confirmed to have other diseases after surgery) were ruled out, and clinical data was collected from the remaining 83 patients for statistical analysis. These patients were divided into an open surgery group (n=62) and a laparoscopic surgery group (n=21) according to the surgical methods used, and after 1:2 propensity score matching (PSM), 32 and 16 patients respectively in the open surgery group and laparoscopic surgery group were remained. The demographic data, Bismuth type, perioperative data, intraoperative data, postoperative complications, pathological findings, and long-term survivals were compared between these two groups.

**Results:**

After 1:2 PSM, 32 patients in the open surgery group and 16 patients in the laparoscopic surgery group were included for further analysis. Baseline characteristics and pathological outcomes were comparable between the two groups. Statistically significant differences between the two groups were observed in intraoperative blood loss and operative time, as it were 400-800 mL vs 200-400 mL (P=0.012) and (407.97 ± 76.06) min vs (489.69 ± 79.17) min (P=0.001) in the open surgery group and laparoscopic surgery group, respectively. The R0 resection rate of the open group was 28 cases (87.5%), and the R0 resection rate of the laparoscopic group was 15 cases (93.75%). The two groups showed no significant difference in terms of surgical approach, intraoperative blood transfusion, incidence of postoperative complications, and short- and long-term efficacy (P>0.05).

**Conclusions:**

Laparoscopic radical resection of HCCA has comparable perioperative safety compared to open surgery group, as it has less bleeding and shorter operation time. Although it is a promising procedure with the improvement of surgical skills and further accumulation of experience, further investigations are warranted before its wider application.

## Introduction

Radical resection remains the most effective treatment for hilar cholangiocarcinoma (HCCA) (1–4), and HCCA patients have a 5-year survival rate of less than 40% (1–3). Due to the complex anatomy of the hilar region and the high incidence of anatomical variations, HCCA is prone to invade portal vein and hepatic arteries, resulting in a low resectable rate and high surgical difficulty (4). In fact, successful laparoscopic radical resection of HCCA requires excellent surgical skills and rich experience. With the improvements in minimally-invasive surgical instruments, surgical skills, and accumulation of surgical experience, more patients have undergone laparoscopic or robotic radical resection of HCCA (5–11). Herein we retrospectively analyzed the clinical data of the patients who underwent radical surgery for HCCA in our center, and compared the efficacy of laparoscopic and open surgery for the patients with HCCA.

## Materials and methods

### Patients

**Figure 1:**
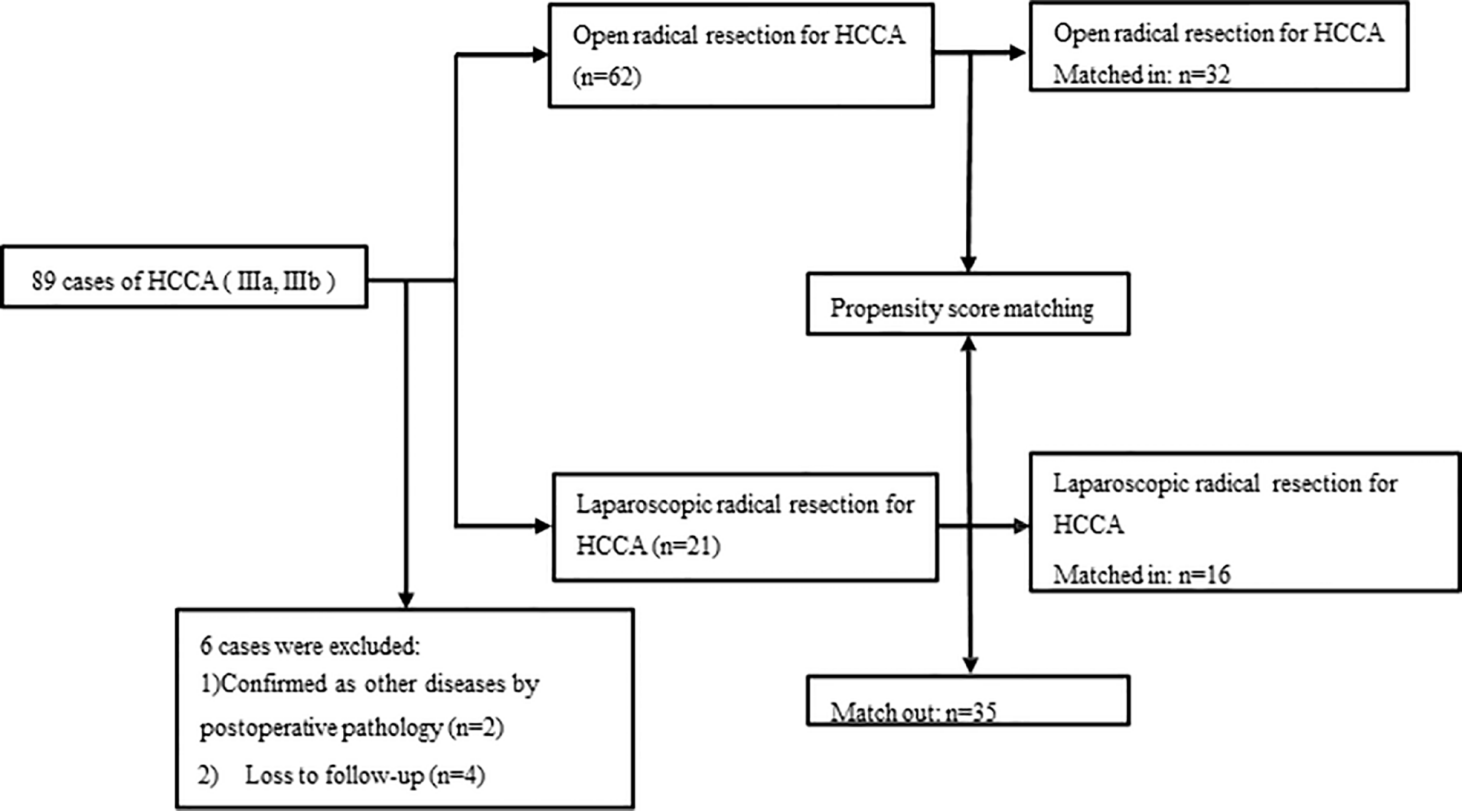
Flow chart of patient enrollment.

We retrospectively analyzed the clinical data of 89 patients with a confirmed diagnosis of HCCA (IIIA, IIIB) by imaging [abdominal ultrasound, computed tomography (CT), and magnetic resonance cholangiopancreatography (MRCP)] and postoperative pathology who underwent radical resection in our center from January 2018 to March 2022. These patients were divided into open surgery (OS) group (n=62) and laparoscopic surgery (LS) group (n=21) according to the surgical modality used, 32 in OS group and 16 in LS group were finally included after 1:2 propensity score matching (PSM) (**Figure 1**). The demographic data, Bismuth type, perioperative data, intraoperative data, postoperative complications, pathological findings, and follow-up outcomes were compared between the two groups. This retrospective observational study was approved by the Medical Ethics Commission of our hospital(2022-r111-01) and was conducted in accordance with the Declaration of Helsinki and the *International Ethical Guidelines for Biomedical Research Involving Human Subjects.*

### Preoperative management

Before the surgery, abdominal ultrasound, multidetector CT (MDCT), and MRCP were routinely performed in all patients to assess the extent of bile duct and blood vessel involvements and to determine whether the tumor was resectable. For resectable tumors, a three-dimensional (3D) visualization system was used to assess the presence (or absence) of anatomical variations in the bile ducts and vessels in the hilar region and calculate the volume of the remnant liver. For patients with suspected lymph node metastasis, PET-CT was further performed to rule out any distant metastasis. For patients with a serum total bilirubin (TBil) level of higher than 100 mmol/L, percutaneous transhepatic biliary drainage (PTBD) was performed to lower TBil level and relieve biliary hypertension.

### Surgical methods

Except for the different surgical approaches, both laparoscopic and open radical resection of HCCA followed the same surgical principles and resection criteria according to the guidelines (12). The scope of resection for Bismuth type III or IV HCCA included extrahepatic bile duct, left (or right) half of the liver, and caudate lobe, along with regional lymph node dissection. Anatomical liver resection was performed. To achieve R0 resection, we routinely resected the tumor and sent the surgical margins of proximal and distal bile duct for intraoperative frozen section analysis.

**Figure 2:**
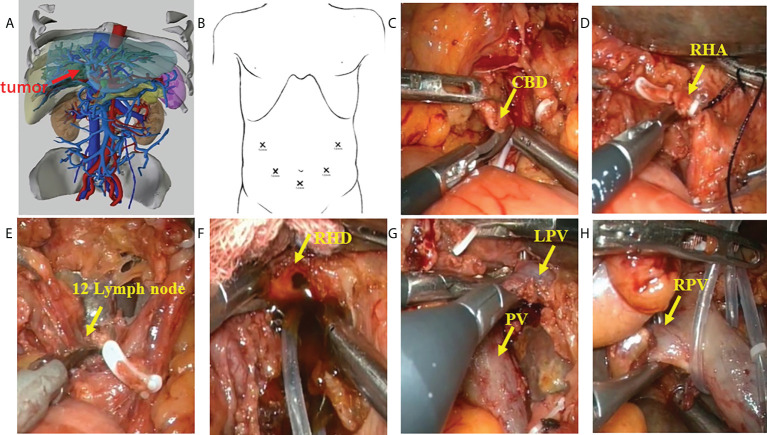
Procedure of laparoscopic hilar cholangiocrinoma 1. **(A)** three-dimensional (3D) imaging of the tumor (red arrow); **(B)** Trocar placement during laparoscopic radical resection for HCCA. The chief operator stands on the right side of the patient, inserting 5-mm and 12-mm trocars into the right abdomen; the first assistant stands on the patient’s left side, placing 5-mm and 12-mm trocars above the umbilicus and on the left abdomen; and the camera-holder stands between the two legs of the patient(yellow arrow). **(C)** sever the lower end of the common bile duct at the upper border of the pancreas; **(D)** transect the right hepatic artery(yellow arrow); **(E)** dissect the lymph nodes in the hilar region(yellow arrow); **(F)** transect the right hepatic duct(yellow arrow); **(G)** identify the left branch of portal vein and portal vein (yellow arrow); **(H)** identify the right branch of portal vein (yellow arrow). CBD, Common bile duct; RHA, Right hepatic artery; RHD, Right hepatic duct; LPV, Left branch of portal vein; PV, Portal vein; RPV, Right branch of portal vein.

**Figure 3:**
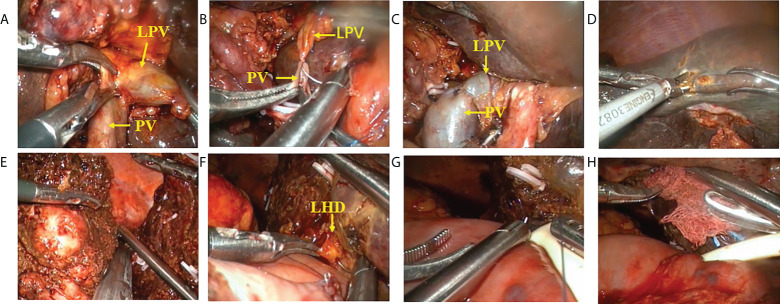
Procedure of laparoscopic hilar cholangiocrinoma 2. **(A–C)** resection and reconstruction of left branch of portal vein; **(D)** liver parenchyma transection-priority approach for liver resection; **(E)** transection of right hepatic vein using a cutter/staple; **(F–H)** hepatobliary duct-jejunum anastomosis (placement of T tube). LPV, Left branch of portal vein; PV, Portal vein; LHD,left hepatic duct.

A careful exploration for ascites and peritoneal/omental metastases was performed first in both groups. Ultrasound was routinely performed to exclude intrahepatic metastases. An inverted L-shaped incision was created in the OS group, and a five-port approach was used in the LS group (**Figures 2A, B**). Patients were fasted for 12 h with water deprivation of 4 h before surgery. The operation steps in the LS group were as follows: 1) The lesser momentum was divided and the liver was suspended to expose the surgical field. 2) At the lower end of the common bile duct and at the upper edge of the pancreas, the surgical margin of lower bile duct margin was obtained for rapid intraoperative pathology. The upper end of the common bile duct was lifted. The lymphs, nerves, adipose tissue, and fibrous connective tissues in the hepatoduodenal ligament were removed during the operation (**Figure 2C**). 3) Stations 8 and 12 lymph nodes were dissected. After the outer sheath of the common hepatic artery was divided, the common hepatic artery was pulled with a thin silicone tube, and the gastroduodenal artery, proper hepatic artery, left and right hepatic arteries, and left and right portal vein branches were separated and skeletonized one after another (**Figures 2D–H**). 4) The Kocher incision was made for dissecting the lymph nodes around the head of the pancreas. 5) After removal of the gallbladder, the left or right hepatic artery and the left or right branch of the portal vein were severed, during which both the proximal and distal ends of the vessels were ligated with 10-gauge sutures, followed by the closure of the distal end with a plastic clip. In case of portal vein involvement, portal vein resection and reconstruction were performed (**Figures 3A–C**). 6) The blood flow into the liver was blocked using laparoscopic bulldog forceps, and intraoperative ultrasound was used to locate the middle hepatic vein, which was marked on the surface of the liver. The extent of liver resection was assessed preoperatively; accordingly, the left-half liver or right-half liver plus caudate lobe was resected *via* hepatic parenchymal transection-priority approach, during which the bile ducts and vessels, if encountered, were clamped using plastic clips or titanium clips and then disconnected. Subsequently, the half liver and caudate lobe were completely resected (**Figures 3D, E**). 7) The hepatobiliary ducts in liver remnant were identified and the surgical margins of the bile ducts were sent for rapid pathology. The hepatobiliary ducts in liver remnant were prepared for hepatobiliary duct-jejunum end-to-side anastomosis. 8) The jejunum was severed 20 cm below the ligament of Treitz. A side-to-side anastomosis was performed 50 cm below the proximal jejunum and distal jejunum, followed by the closure of the mesangial foramen. The distal jejunum and the colon were lifted anterosuperiorly for end-to-side Roux-en-Y hepaticojejunostomy with the bile duct in liver remnant, and biliary drainage tube was placed during the surgery in some patients (**Figures 3F–H**). 9) Abdominal drainage tubes were placed near the anastomosis site and liver section, respectively. Finally, the resected specimens and lymph nodes were sent for histopathological examinations.

### Postoperative management

After the surgery, the patients were closely monitored in the surgical intensive care unit. Patients were given total parenteral nutrition before oral intake, according to the advice of the nutrition department. Prophylactic antibiotics, proton pump inhibitors (PPIs), and liver-protecting drugs were routinely administered. Generally, patients started a liquid diet on the postoperative day 3. The abdominal drainage volume was observed, and the possible bleeding or biliary fistula was evaluated. On day 5, all patients were re-examined with abdominal plain CT to identify whether there was ascites, and the abdominal drainage tube was taken out based on CT findings, color of drainage fluid, and inflammatory markers. In our center, the drainage tube was usually removed 6 - 8 days after operation.

Chemotherapy with gemcitabine or gemcitabine combined with cisplatin was recommended after discharge. All patients chose their chemotherapy protocols based upon their own willingness.

### PSM

PSM is a useful statistical method for pre-processing data from observational studies and are widely used in retrospective studies to reduce the effects of confounding variables and other sources of bias, thus allowing for more reasonable comparisons between observational and control groups (13). In the present study, the LS and OS groups were compared using a 1:2 PSM to minimize differences among patient populations. Due to differences in baseline data, Logistic regression was used to calculate the propensity score of each patient; after 1:2 nearest neighbor matching, patients who did not meet the matching criteria were excluded.

### Definitions

The common complications after radical resection of HCCA include intra-abdominal hemorrhage, stress ulcer bleeding, liver failure, ascites complicated with infection, bile leakage, biliary-enteric anastomotic stenosis, and delayed gastric emptying (DGE). The diagnosis of surgical site infection (SSI) was based on the criteria developed by the National Nosocomial Infections Surveillance System (NNIS), US Centers for Disease Control (14). The diagnosis of DGE was based on the definition suggested by the International Study Group of Pancreatic Surgery (ISGPS) in 2007 (15), i.e., a diagnosis of DGE can be made if one of the following conditions occurs after excluding mechanical factors such as anastomotic obstruction by upper gastrointestinal barium study or gastroscopy: a) the gastric tube needs to be indwelled for more than three days after surgery; b) the gastric tube needs to be re-inserted due to vomiting and other reasons after extubation; and c) solid food is still not allowed seven days after surgery. The short-term postoperative complications were graded using the 2004 Clavien-Dindo system (16). TNM staging was based on the eighth edition of the American Joint Committee on Cancer staging manual and tumor anatomic type was classified according to the Bismuth-Corlette system (17, 18).

### Statistical analysis

Statistical analysis was performed using the SPSS 26.0 software package (SPSS Inc., IBM, Armonk, NY). The measurement data were first tested for normality and homogeneity of variance. The normally distributed or homogenous measurement data are presented using mean ± standard deviations and analyzed using t test or Chi-square test, otherwise they are presented using the medians (interquartile range) and analyzed using rank sum test. The count data are presented by the number of cases (percentage) and were analyzed using the Chi-square test, Chi-square test with continuity correction, or Fisher’s exact test when appropriate. The Kaplan-Meier survival curves were used to compare the overall survival (OS) and disease-free survival (DFS) between these two groups. During PSM, Age, BMI, History of abdominal surgery, PTBD, ASA score, Bismuth type, and tumor diameter were used as covariates. the nearest neighbor matching method was used for 1:2 matching, and the caliper value was 0.1. All P values reported were two-tailed and a P value of <0.05 was considered significantly different.

## Results

A total of 89 patients who had HCCA (IIIA, IIIB)and undergone radical HCCA resection were analyzed in this study, however six patients were excluded due to 1) confirmed as other diseases by postoperative pathology (n=2) and 2) lost to follow-up (n=4). Finally, 83 patients were included in this study, including 21 patients in the LS group and 62 patients in the OS group. After 1:2 PSM, 32 and 16 patients respectively in the OS and LS group were selected for further comparative analysis. We searched hospital electronic medical records to extract patient information, including demographic features, comorbidities, preoperative blood and imaging studies, tumor characteristics, intraoperative data, and postoperative data. Patients were followed up by phone or outpatient visits. Tumor recurrence and deaths were recorded.

### Preoperative data

The preoperative data and pathological results of all patients are shown in **Tables 1** , **2**, respectively. There were no differences in terms of gender, age, body mass index (BMI), American Society of Anesthesiology physical status (PS) score, disease status, percutaneous transhepatic biliary drainage (PTBD), drinking and smoking histories, underlying diseases, biochemical tests, and history of abdominal surgery between the two groups (*P*>0.05). The Bismuth type, diameter, pathological differentiation, TNM stage, nerve invasion, microvascular invasion of the tumors, as well as the number of cleared lymph node and positive lymph nodes all showed no significant differences between these two groups (all P>0.05).

**Table 1:** Demographic and baseline characteristics in the OS and LS groups.

Variables	OS group (n = 32)	LS group (n = 16)	*P* value
Age [median (q1-q3), years]	62.5(52.25-67)	64(54-66)	0.991
BMI[mean ± SD,kg/m^2^]	22.7 ± 2.71	23.54 ± 2.45	0.300
Gender, n (%)			0.683
Female	16 (50)	9 (56.25)	
Male	16 (50)	7 (43.75)	
ASA score, n (%)			0.781
1	20 (62.5)	9 (56.25)	
2	9(28.12)	6 (37.5)	
3	3 (9.38)	1 (6.25)	
Underlying diseases (heart disease, lung disease, diabetes, etc.), n (%)			0.911*
None	23 (71.88)	11 (68.75)	
Yes	9 (28.12)	5 (31.25)	
Smoking, n(%)	7 (21.88)	2 (12.5)	0.695*
Drinking, n (%)	5 (15.63)	2 (12.5)	0.885*
PTBD, n (%)	21 (65.63)	12(75)	0.509*
History of abdominal surgery, n (%)	5 (15.63)	4 (21.05)	0.885*
Biochemistry			
CA19-9[median (q1-q3), U/ml]	150.66(16.09-800)	135.74(50.94-587.83)	0.775
CEA[median (q1-q3), ng/ml]	4.13(2.2-6.26)	2.45(1.94-3.95)	0.094
CA125[median (q1-q3), U/ml]	18.95(11.8-28.5)	15.9(10.5-26.2)	0.548
AST[median (q1-q3), U/L]	95.9(55.28-167.53)	71.6(49.8-151.4)	0.484
ALT [median (q1-q3), U/L]	140.85(61.55-232.10)	123.00(43.00-180.50)	0.217
TBil[median (q1-q3), umol/L]	203.10(93.43-373.20)	227.00(87.30-317.40)	0.687
<34.2	6 (18.75)	1 (6.25)	0.470*
≥34.2	26 (81.25)	15 (93.75)	

Data are presented as standard deviation (mean ± SD), or as median (interquartile range), or as number (percentage). OS: Open Surgery; LS: Laparoscopic Surgery; BMI, Body Mass Index; ASA, American Society of Anesthesiologists; PTBD, Percutaneous Transhepatic Biliary Drainage; CA-199, Carbohydrate antigen 19-9; CEA, Carcinoembryonic antigen; CA125, Carcinoembryonic antigen 125; ALT, Alanine aminotranferease; AST, Aspartate aminotransferase; TBil, Total Bilirubin. *Fisher exact test.

**Table 2:** Pathological findings in the OS and LS groups.

Variables	OS group (n = 32)	LS group (n = 16)	*P* value
Bismuth type, n (%)			0.838
IIIa	15 (46.88)	7 (43.75)	
IIIb	17(53.13)	9 (56.25)	
Tumor diameter [mean ± SD,cm]	2.66 ± 1.04	2.58 ± 1.13	0.697
Degree of differentiation, n (%)			0.402
Well-differentiated	5 (15.63)	1 (6.25)	
Moderately-differentiated	12 (37.5)	9 (56.25)	
Poorly-differentiated	15 (46.88)	6 (37.5)	
TNM stage, n (%)			0.956
I	1 (3.12)	1 (6.25)	
II	8 (25)	4 (25)	
IIIA	6 (15.79)	2 (12.5)	
IIIB	6 (18.75)	52(12.5)	
IIIC	7 (21.88)	4 (25)	
IVA	4 (12.5)	3 (18.75)	
Perineural involvement, n (%)	11 (34.38)	6 (37.5)	0.831
Microvascular invasion, n (%)	1 (3.12)	1 (6.25)	0.798*
Lymph node involvement, n (%)			
Total number [median (q1-q3)]	6 (5 - 7)	7 (5 - 8)	0.146
Positive rate	7 (21.88)	7 (43.75)	0.217*

Data are presented as standard deviation (mean ± SD) or as median (interquartile range). * Fisher’s exact test. OS, open surgery; LS, laparoscopic surgery.

### Intraoperative and postoperative data

The intraoperative and postoperative data are shown in **Table 3**. All surgeries were completed under laparoscope and none of them was converted to open surgery in LS group. The two groups showed no significant differences in terms of surgical approach, intraoperative blood transfusion, and incidence of postoperative complications (*P*>0.05). The intraoperative blood loss, operative time in the OS group and LS group had statistically significant differences which、 were 400-800) vs (200–400) mL (P =0.012), and (407.97 ± 76.06)min vs (489.69 ± 79.17)min (P=0.001), respectively.

**Table 3:** Intraoperative data and surgical effectiveness in the OS and LS groups.

Variables	OS group (n = 32)	LS group (n = 16)	*P* value
Operative time (mean ± SD, min)	407.97 ± 76.06	489.69 ± 79.17	0.001
Blood loss [median (q1-q3), mL]	600(400-800)	300(200-400)	0.012
Intraoperative blood transfusion, n (%)	24(75)	8(50)	0.083
Hepatectomy			0.838
Left hemihepatectomy + hepatectomy, n (%)	17 (53.12)	13 (56.25)	
Right hemihepatectomy + hepatectomy, n (%)	15 (46.88)	6 (43.75)	
Resection margin, n (%)			0.867*
R0	28 (87.5)	15 (93.75)	
R1	4 (12.5)	1 (6.25)	
Vascular resection and reconstruction, n (%)	2 (6.25)	1 (6.25)	0.527*
Complications, n (%)			
Clavien-Dindo grade < 3	25 (78.12)	14(87.5)	0.695*
Clavien-Dindo grade ≥ 3	7(21.88)	2(12.5)	
Incision infection	1 (3.12)	1 (6.25)	0.798*
Gastrointestinal hemorrhage	0 (0)	0 (0)	1.000*
Intra-abdominal hemorrhage	1(3.13)	0 (0)	0.721*
Delayed gastric emptying	2 (6.25)	1 (6.25)	0.527*
Pleural effusion	3 (9.38)	1 (6.25)	0.854*
Ascites	2 (6.25)	1 (6.25)	0.527*
Pulmonary infection	3 (9.38)	2 (12.5)	0.867*
Abdominal infection	2 (6.25)	1 (6.25)	0.527*
Liver failure	0 (0)	0 (0)	1.000*
Bile leakage	0 (0)	0 (0)	1.000*
Post-operative hospital stay [median (q1-q3),day]	14(11.25-21.25)	11.5(10.00-17.75)	0.254*
30-day readmission rate, n (%)	4 (12.5)	1 (6.25)	0.867*
90-day mortality rate, n (%)	1 (3.12)	0 (0)	0.721*
Hospitalization expenses [median (q1-q3),RMB]	95697(80306.25-117588.33)	105170(98160.05-119130)	0.213

Data are presented as standard deviation (mean ± SD) or as number (percentage). * Fisher’s exact test. OS, open surgery; LS, laparoscopic surgery.

Incision infection occurred in one patient in each group, which was improved after intensive dressing changes. In the OS group, one patient suffered from intra-abdominal hemorrhage, which was relieved after treatments such as cryoprecipitate infusion, improvement of coagulation function, and blood transfusion. Delayed gastric emptying (DEG, also known as gastroparesis) occurred in two patients in OS group and one patients in LS group, they were treated with gastrointestinal decompression, enhanced nutrition, and gastrokinetic drugs. In the OS group, two patients suffered from peritoneal effusion accompanied by intra-abdominal infection, which were improved after the placement of peritoneal catheter and the use of antibiotics; and three patients had pleural effusion, of whom one patient had pulmonary infection and was cured by thoracentesis catheter drainage and antibiotic treatment, and the other two patients were improved after drug adjustment. In the OS group and LS group, no patients developed liver failure or biliary leakage. In the LS group, one patients experienced pleural effusion accompanied by pulmonary infection, which was improved after medicinal treatment and functional training, and the other pulmonary infection patients were improved after drug adjustment; 1 patient suffered from intra-abdominal infection accompanied by ascites, which was improved after catheter drainage and antibiotic use (based on the results of bacterial culture). The hospitalization cost were 95697 (80306.25-117588.33) RMB versus 105170 (98160.05-119130) RMB and the postoperative hospital stay were 14(11.25-21.25) days versus 11.5(10.00-17.75) days respectively in the OS group and the LS group, with no statistically significant differences (*P*>0.05). One patient in the OS group died of gastrointestinal bleeding 2 months after operation. There was no statistically significant difference in readmission within 30 postoperative days (*P*>0.05).

### Long-term outcomes

**Figure 4:**
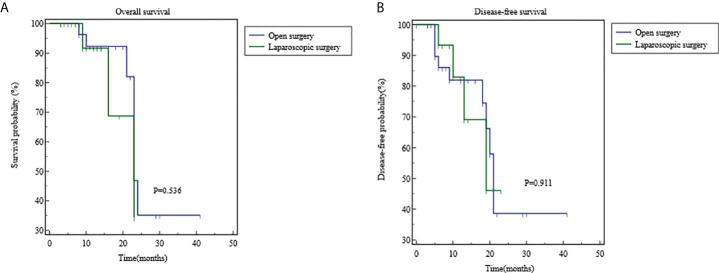
Comparisons of overall survival and disease-free survival using Kaplan-Meier curves. **(A)** the 1- year survival rates were 92.28% in the OS group and 91.67% in the LS group, and 2-year survival rates was 35.16% in the OS group and 34.37% in the LS group (P=0.536). **(B)** The 1-year disease-free survival (DFS) rate was 82.16% in the OS group and 82.96% in the LS group, and the 2-year DFS rate was 38.64% in the OS group and 46.09% in the LS group (P=0.911).

The long-term efficacy in the OS group and the LS group is shown in **Table 4** and **Figure 4**. The median follow-up duration was 13.5 months in the OS group and 12 months in the LS group (P=0.303). Recurrence was noted in 4 cases (25%) in LS group, including three case of local recurrence (18.75%) and one case of distal metastasis (6.25%); in the OS group, ten cases (31.25%) progressed including five case of local recurrence (15.63%), and five cases of distal metastasis (15.63%). There was no statistical difference in the total recurrence rate, local recurrence rate, and distant metastasis rate between these two groups (P>0.05). During the follow-up period, seven patients (21.88%) in the OS group and three (18.75%) patient in the LS group died due to disease progression (P=0.999). The 1- year survival rates were 92.28% in the OS group and 91.67% in the LS group, and 2-year survival rates was 35.16% in the OS group and 34.37% in the LS group (P=0.536).The 1-year disease-free survival (DFS) rate was 82.16% in the OS group and 82.96% in the LS group, and the 2-year DFS rate was 38.64% in the OS group and 46.09% in the LS group (P=0.911).

**Table 4:** Long-term outcomes in the OS and LS groups.

Variables	OS group (n = 32)	LS group (n = 16)	*P* value
Followed-up duration [median (q1-q3), months]	13.5 (9.25_21.75)	12 (8.25 – 15.50)	0.303
Total recurrence rate, n (%)	10(31.25)	4 (25)	0.911*
Local recurrence	5 (15.63)	3 (18.75)	0.999*
Distant metastasis	5 (15.63)	1(6.25)	0.643*
Total mortality rate, n (%)	7 (21.88)	3 (18.75)	0.999*

^#^Wilcoxon signed rank test. *Chi-square test with continuous correction. OS, open surgery; LS, laparoscopic surgery.

## Discussion

HCCA is an extremely destructive tumor that is difficult to diagnose and responds poorly to radiotherapy and chemotherapy. Complete tumor resection iscrucial for the long-term survival of patients with HCCA, in whom the 5-year survival rate is below 40% (1–4). However, due to the complex anatomy of the perihilar region and the high incidence of anatomical variations, HCCA is prone to invade the adjacent vessels, liver parenchyma, and pancreas, showing unique biological features. Thus, surgical treatment of HCCA is highly challenging (4). In recent years, laparoscopic technology has increasingly been applied in complex upper abdominal operations such as hepatectomy, radical gastrectomy, and pancreaticoduodenectomy, offering strong technical support and experience for laparoscopic radical resection of HCCA (19–22).

Since YU et al. (23) for the first time described the successful laparoscopic radical resection of HCCA in 2001, more similar cases have been reported. However, none of these reports involved the hemi-hepatectomy combined with caudate lobectomy. Gumbs et al. (24) reported minimally invasive treatment of extra-pancreatic cholangiocarcinoma, including 5 cases of minimally invasive resection of hilar cholangiocarcinoma, and 2 cases of laparoscopic extensive hepatectomy, with and postoperative recovery, achieving good curative effect.

Lee et al. (25) reported laparoscopic resection of HCCA in five patients, three of whom underwent hemihepatectomy combined with caudate lobectomy. In 2018, Zhang et al. (26)reported a similar case. However, all these were published in the form of case report, and did not compare the postoperative outcome. In 2019, Zhang et al. compared the performance of open versus laparoscopic radical resection in treating HCCA (6). It was found that there were no significant differences in postoperative hospital stay, blood loss, blood transfusion rate, and complications between these two groups. However, the laparoscopic group had significantly longer operative time as well as lower 1- and 2-year survival rates. Ratti et al. (27) compared the clinical data of HCCA patients undergoing laparoscopic or open radical resection and found that the laparoscopic group had less intraoperative blood loss, lower intraoperative blood transfusion rate, and shorter postoperative hospital stay than the open surgery group but with longer operative time; and there was no significant difference in R0 resection rate and postoperative survival time between these two groups. In 2021, Ma et al. (5) compared the laparoscopic versus open resection in HCCA patients and found that, in terms of long-term prognosis, the OS and DFS rates of the open surgery group were significantly higher than those of the laparoscopic group; however, the difference in the followed-up period between these two groups was statistically significant. The above reports are a comprehensive comparison of type I, II, III and open group, without separate clinical observation of laparoscopic and open surgery for type III. Laparoscopic completion of type I and type II is not controversial, and the operation is not difficult, but for type III, it is still controversial whether laparoscopy can be completed because of the difficulty of operation. This is a retrospective study on type III, having a large number of cases of type III, thus with more valuable observable results. It is found that compared with laparotomy, laparoscopy has no differences in the postoperative complications and postoperative survival rate. Moreover, its bleeding is lower than laparotomy. So, the laparoscopic surgery is safe and feasible, which is not wholly consistent with Ma et al. (5) report. The reason may be that the surgeon has experience in LPD500 cases, most of which were completed after 2019, achieving more R0 resection. Therefore, we primarily present our experience that having 150 cases of LPD and 50 cases of laparoscopic hemi-hepatectomy is the basic requirement for laparoscopic hilar cholangiocarcinoma, which can ensure not inferior to the laparotomy surgery complication.

In our retrospective observation, the operation time of the laparoscopic group was prolonged, which was statistically significant in terms of the comparison between the two groups. But it is expected that similar to LPD, this difference will be significantly shortened with the increase of proficiency. In addition, Sucandy I et al. (28) reported that 15 patients who underwent robot-assisted radical resection of hilar cholangiocarcinoma recommended that robotic technology should be considered as an alternative to “open resection”. Admittedly, robot has been widely used in liver surgery because of many advantages (29), and it is also one of the promising options for minimally invasive treatment of hilar cholangiocarcinoma. Although it is limited by its high price in China, we expect that it will be more widely used in the future.

Studies (30–32) have shown that R0 resection is the most important factor to achieve long-term survival in patients with HCCA, and a positive resection margin directly affects the prognosis of the patients. R0 resection requires negative surgical margins in multiple structures such as bile duct, liver, and blood vessels.

Tsao et al. (33) and Kow et al. (34–38) reported that the combination with caudate lobectomy raised the R0 resection rate and prolonged patient survival. From a pathological perspective, Nimura et al. (32)also concluded that resection of the caudate lobe could benefit patients in long-term survival. At present, routine hemihepatectomy combined with caudate lobectomy has been widely recommended in the radical resection of HCCA (36–38). Unfortunately, most literature on the hemihepatectomy combined with caudate lobectomy for HCCA were retrospective studies (34–38) and therefore their findings were inevitably subjected to confounding factors. In the present study, the use of PSM enabled the comparability of the general data between two groups and increased the reliability of our findings.

In addition, although the preoperative assessment can improve our initial judgment of resectability, the final judgment needs to be made by the operator after intraoperative exploration. In cases where intraoperative exploration reveals vascular invasion on the side scheduled to be preserved and R0 may be achieved by the combined resection, hemihepatectomy combined with caudate lobectomy along with vascular resection and reconstruction may be performed (39, 40). In our series, portal vein involvement was found in one patient in each group, and a negative vascular margin was achieved after portal vein resection and reconstruction during the surgery. According to our experience, such operation can be done by experienced operators in large centers; if laparoscopic vascular resection and reconstruction is difficult to perform, timely intraoperative conversion to laparotomy is required to ensure surgical safety. Since laparoscopic hepatic artery resection and reconstruction is highly challenging and risky, along with questionable quality of the anastomosis, it should be carried out with caution (39).

Based on the R0 resection, standardized regional lymph node dissection is another important factor to ensure the long-term survival of patients with HCCA (41). Research has suggested that lymph node metastasis is an independent risk factor affecting the prognosis of patients with HCCA, and regional lymph node metastasis is a key predictor (41). Therefore, lymph node dissection in the perihilar region is a critical step in radical resection. However, due to the diverse techniques and concepts of radical resection among different medical centers, the optimal number of lymph nodes to be dissected also differs; accordingly, the optimal number of regional lymph nodes to be dissected is also inconclusive (42–44). According to our experience, at least 5 lymph nodes need to be dissected during the radical resection of HCCA, and the dissection range should include the lymph nodes and nerve plexus tissues in hepatoduodenal ligament, near the common hepatic artery, and behind the head of the pancreas. All of these tissues except the hepatic artery and portal vein must be resected to achieve the skeletonized dissection. In the present study, there was no statistical difference in the total number of dissected lymph nodes between the LS group and the OS group.

Notably, peripheral blood vessels should be carefully protected during lymph node dissection. According to Zhang et al. (6), excessive dissection of lymph nodes around the hepatic artery resulted in mechanical damage to the blood vessels, leading to postoperative pseudoaneurysm of the hepatic artery. With the maturity of laparoscopic liver resection and pancreaticoduodenectomy, laparoscopic hilar lymph node dissection has increasingly been applied. Using intrathecal separation and dissection techniques, the laparoscopic procedure minimizes the direct clamping of blood vessels with surgical instruments. Preferably, a vessel loop is used to suspend and stretch the vessel, so as to minimize the damage to the intima of the arteries and prevent serious complications such as postoperative aneurysm. If lymph node station 13 is found to be positive by intraoperative rapid pathology, station 16 should be dissected and sent for rapid pathology. If the result is also positive, radical resection should be abandoned.

In the present study, there was no statistically significant difference in perioperative safety between the LS and the OS group. We believe that laparoscopic radical resection of HCCA will be increasingly adopted with the improvements in surgical skills and accumulation of experience. However, it remains a challenging and high-risk technique in its initial stage and should be performed only in carefully selected patients in large hepatobiliarypancreatic surgery centers. With patient safety as the top priority, the surgical procedures should be standardized to ensure surgical safety and prolong the long-term survival.

Based on our experience, Surgical indications of laparoscopic radical resection for hilar cholangiocarcinoma were as follows: 　① hilar cholangiocarcinoma(I, II) and part hilar cholangiocarcinoma (IIIa, IIIb)(no invasion to points U or P, preferably no invasion to the secondary bile duct branches) and sufficient residual liver volume after tumor resection was clearly diagnosed without signs of distant metastasis based on preoperative imaging and biochemical tests; ②the tumor did not invade key peripheral blood vessels such as the portal vein and hepatic artery and did not require combined vascular resection; ③no severe multiple organ dysfunction such as heart, lung, kidney and brain, or combined with underlying diseases can tolerate surgery after active adjustment. In our experience, if there are both more than 10 cases of experience in laparoscopic pancreaticoduodenectomy combined with vascular resection and reconstruction and more than 10 cases of laparoscopic radical resection of hilar cholangiocarcinoma, the hilar cholangiocarcinoma combined with vascular resection and reconstruction can be tried.

Our study had some limitations. First, as a retrospective study, it lacked prospective design and randomization. Although PSM was used, it could not fully rule out the confounding factors, and there were certain biases. Second, the sample size was small. Thus, prospective multi-center clinical studies with large sample sizes are needed to further validate the safety and effectiveness of laparoscopic radical resection of HCCA and standardize its surgical steps.

## Conclusion

In summary, this retrospective observational analysis demonstrated that laparoscopic radical resection of HCCA is safe in the perioperative period and can be performed in large hepatobiliarypancreatic surgery centers after careful assessment. Our results showed that the efficacy of LS group was comparable to that of OS group, we are confident that the long-term efficacy of this technique will be dramatically improved with the improvements in surgical skills, accumulation of experience and prolonged follow-up period.

## Data availability statement

The original contributions presented in the study are included in the article/**Supplementary Material**. Further inquiries can be directed to the corresponding authors.

## Ethics statement

Written informed consent was obtained from the patient for publication of this report and any accompanying images.

## Author contributions

Y-GH, Y-ML, X-BH and LZ contributed to conception and design of the study, and drafted the manuscript. JL, F-XY, L-QL, X-HP, WH, C-LD and Y-CT contributed to analysis and interpretation of data and revised the manuscript. WH, QR and Y-GH participated in clinical treatment operation and literature research. All authors read and approved the final manuscript.

## Funding

This study was supported by grants from the Chongqing Municipal Science and Technology Innovation Project for Social Undertakings and People’s Livelihood Guarantee (cstc2018jscx-mszdX0012); and the Natural Science Foundation of Chongqing (cstc2019jcyj-msxmX0498 and cstc2021jcyj-msxmX0991).

## Conflict of interest

The authors declare that the research was conducted in the absence of any commercial or financial relationships that could be construed as a potential conflict of interest.

## Publisher’s note

All claims expressed in this article are solely those of the authors and do not necessarily represent those of their affiliated organizations, or those of the publisher, the editors and the reviewers. Any product that may be evaluated in this article, or claim that may be made by its manufacturer, is not guaranteed or endorsed by the publisher.
